# Biological Activities and Potential Applications of Phytotoxins

**DOI:** 10.3390/toxins16100444

**Published:** 2024-10-16

**Authors:** Marco Masi

**Affiliations:** Department of Chemical Sciences, Complesso Universitario Monte Sant’Angelo, University of Naples, Federico II, Via Cintia 4, 80126 Napoli, Italy; marco.masi@unina.it

Specialized metabolites, also known as secondary metabolites, produced by plants and microbes possess several biological activities [1]. Among them, phytotoxins are substances which are poisonous or toxic for plants [2]. They seem to play an important role in plant–pathogen interactions when produced by phytopathogenic fungi or bacteria, while plant-produced phytotoxins—including allelochemicals—seem to be involved in plant–plant interactions [3]. These compounds belong to different classes of natural substances and the determination of their chemical structure, or their identification, are fundamental for understanding their function [4]. Most of these phytotoxins have shown interesting potential applications in agriculture or medicine [5]. However, many biological activities of these compounds have not been investigated, limiting their potential practical use. They can serve as efficient tools to design safe biopesticides, avoiding the use of synthetic pesticides that are able to cause long-term impact of residues in agricultural products with a risk to human and animal health [3,6]. They can also be used for the development of new, more effective drugs to overcome problems of antibiotic resistance being the reservoir of new mechanisms of action [7]. 

This Special Issue is focused on the isolation and characterization of new bioactive phytotoxins or on the evaluation of the biological activities of known phytotoxins to further investigate their potential applications in different fields. This collection comprises eleven papers with eight research articles [Contributions 1–8] and three reviews [Contributions 9–11].

Some research articles investigated the production of phytotoxins by plants as potentialbiopesticides for the control of parasitic plants and fungal phytopathogens that can cause devastating losses to crop yields in several parts of the world [Contributions 1–2]. Among them, *Orobanche cumana* is an obligate holoparasitic plant with strong effects in sunflower crops, while the fungus *Stemphylium vesicarium* has pathogenic effects on many hosts, especially on pear. In the first research article [Contributions 1], the authors investigated the production of specialized metabolites from the allelopathic plant *Retama raetam* and their activity against *S. vesicarium* and *O. cumana*. Six compounds belonging to isoflavones and flavones subgroups have been isolated from the *R. raetam* dichloromethane extract and identified using spectroscopic and optical methods. Among them, laburnetin and ephedroidin resulted in being the most promising metabolites for the control of these pests [Contributions 1]. The allelopathic effects of specialized metabolites produced by the plant *Bellardia trixago* on the growth of *O. cumana* seedlings were described in the second research article contribution [Contributions 2]. Five iridoid glycosides were isolated together with benzoic acid from the ethyl acetate extract of aerial green organs of this plant by bio-guided purification. Among them, melampyroside was found to be the most abundant constituent in the extract (44.3% *w*/*w*), as well as the most phytotoxic iridoid on *O. cumana* radicle, showing a 72.6% inhibition in radicle growth. The ecotoxicological profile of melampyroside was evaluated, providing useful information for the generation of green bioherbicides for the control of parasitic plants [Contributions 2].

Phytotoxins produced by phytopathogenic fungi were also investigated. The third article contribution to the Special Issue [Contributions 3] reports a comparative analysis of secondary metabolites produced by the phytopathogenic fungus *Ascochyta fabae* under in vitro conditions and their phytotoxicity on the primary host, *Vicia faba*, and related legume crops. The produced metabolites were analyzed by NMR and LC-HRMS methods, resulting in the dereplication of seven metabolites, which varied with cultural substrates. The phytotoxicity of the pure metabolites was assessed at different concentrations on their primary hosts and related legumes. Among them, ascosalitoxin and benzoic acid resulted in being the most phytotoxic, and thus are expected to play an important role in necrosis’ appearance [Contributions 3]. Fusaric acid (FA) is one of the first secondary metabolites isolated from phytopathogenic fungi belonging to the genus *Fusarium*, and the fourth article contribution [Contributions 4] provides novel insights on this matter. Specifically, the authors evaluated the effect of different nitrogen sources, iron content, extracellular pH and cellular signaling pathways on the production of FA siderophores by the pathogen *Fusarium oxysporum* [Contributions 4].

The antiviral, anticancer, anti-inflammatory and antibiotic properties of some fungal and plant phytotoxins were also studied. In particular, the fifth research article contribution [Contributions 5] to this Special Issue focuses on the evaluation of natural sesquiterpenes and benzoxazinoids, along with their easily accessible derivatives, against the main protease, RNA replicase and spike glycoprotein of SARS-CoV-2 by molecular docking [Contributions 5]. The sixth research article contribution [Contribution 6] investigates the activity of harzianic acid, a bioactive metabolite derived from fungi of the genus *Trichoderma*, against the Gram-positive bacterium *Staphylococcus aureus* and its role in calcium regulation [Contribution 6]. Naphthoquinones are a valuable source of secondary metabolites that have been well known for their dye properties since ancient times. In the seventh research article contribution [Contribution 7], the authors investigated the biological activity of some naphthoquinone’s derivatives in the search of anticancer lead compounds. Their results encourage further studies on the development of new anticancer drugs for more directed therapies and reduced side effects with the naphthoquinone skeleton [Contribution 7]. Sesquiterpene lactones (SLs) are plant-derived metabolites with a broad spectrum of biological effects, including anti-tumor and anti-inflammatory effects, and are thus promising candidates for drug development. In their herein-presented work, the eighth article contribution [Contribution 8] to this Special Issue, the authors evaluated the effects of selected SLs (grosheimin, costunolide, and three cyclocostunolides) on primary cilia biogenesis and stability in human retinal pigment epithelial (RPE) cells [Contribution 8].

The first review contribution [Contribution 9] provides an overview on the bioactive metabolite production in the genus *Pyrenophora* (Pleosporaceae, Pleosporales). The toxic metabolites discovered from three well-studied species (namely, *P. tritici-repentis*, *P. teres* and *P. semeniperda*) are presented, along with a few reports from additional species, and their isolation, structure determination, and biological activities are discussed [Contribution 9]. The second review [Contribution 10] is focused on momilactones A and B, two labdane-related diterpenoids, mainly identified in rice and several other *Poaceae* species. Their functions, biosynthesis, induction and occurrence in plant species are reported [Contribution 10]. The third review [Contribution 11] is related to the chemistry and biology of piperine. This plant-derived piperamide, isolated from black pepper (*Piper nigrum* L.), possesses several biological activities and potential applications in different fields. The recent progress in its studies, mechanisms of action and structural modifications are summarized to pave the way for future development and utilization of this compound and its derivatives as potent drugs and pesticides [Contribution 11].
